# Predictive Discriminant Validity of the 20-Item Toronto Alexithymia Scale: Incremental Prediction of Emotion Regulation Beyond Psychological Distress

**DOI:** 10.3390/bs16060854

**Published:** 2026-05-27

**Authors:** Ardeshir Mortezaei, Cheyenne S. McIntyre, Graeme J. Taylor, R. Michael Bagby

**Affiliations:** 1 Department of Psychology, D’Youville University, Buffalo, NY 14201, USA; mortea12@dyc.edu; 2 Department of Psychological Science, Ball State University, Muncie, IN 47306, USA; cheyenne.mcintyre@bsu.edu; 3 Department of Psychiatry, University of Toronto, 250 College Street, Toronto, ON M5T1R8, Canada; graeme.taylor@utoronto.ca; 4 Department of Psychology, University of Toronto, 1265 Military Trail, Toronto, ON M1C1A4, Canada

**Keywords:** alexithymia, Toronto Alexithymia Scale, discriminant validity, incremental validity, emotion regulation, psychological distress

## Abstract

Several factor analytic investigations question whether the 20-item Toronto Alexithymia Scale (TAS-20) discriminates alexithymia from general psychological distress, with some researchers concluding that the difficulty identifying feelings (DIF) subscale measures distress rather than alexithymia. This debate has focused almost exclusively on structural discriminant validity (i.e., factor analytic analyses) while neglecting predictive discriminant validity, or whether alexithymia predicts clinically relevant outcomes relatively independent of distress. The present study addressed this issue using hierarchical multiple regression in a community sample enriched with participants having psychiatric histories (N = 661). Emotion regulation was assessed across three domains: global emotion dysregulation (DERS), maladaptive and adaptive cognitive strategies (CERQ), and behavioral strategies (BERQ). After controlling for depression, anxiety, and stress (DASS-21), the TAS-20—entered as its three subscales (DIF, DDF, EOT)—retained statistically significant incremental prediction across all five emotion regulation outcomes. Increments were largest for global emotion dysregulation (ΔR^2^ = 0.11) and behavioral regulation (ΔR^2^ = 0.10 adaptive, 0.09 maladaptive), and smallest for cognitive regulation outcomes (ΔR^2^ = 0.02 adaptive, 0.03 maladaptive); none reached the 0.15 heuristic for a clinically meaningful incremental validity, but all were statistically reliable and obtained against a deliberately conservative test in which alexithymia–distress overlap was partialed prior to entry. Among the subscales, difficulty describing feelings (DDF) and externally oriented thinking (EOT) emerged as the most consistent unique predictors across outcomes after distress control, whereas DIF showed greater attenuation. Notably, a domain-level dissociation emerged whereby alexithymia was the stronger unique predictor of behavioral regulation outcomes while distress showed comparatively greater unique prediction of cognitive strategies, suggesting the two constructs contribute to emotion regulation impairment through partially distinct pathways. These findings support the predictive discriminant validity of the TAS-20: the instrument captures incremental variance in emotion regulation that is statistically reliable across domains and theoretically coherent in its facet-level patterning, even as the construct shows some understandable overlap with concurrent psychological distress.

## 1. Introduction

Alexithymia is a multidimensional personality trait characterized by difficulties identifying and describing emotional feelings, an externally oriented, stimulus-bound thinking style, and constricted imaginal processes (Nemiah et al., 1976; Sifneos, 1973; Taylor et al., 1997). Originally conceptualized through clinical observations of patients with “classical” psychosomatic diseases, alexithymia reflects a fundamental deficit in the cognitive processing and regulation of emotions that has since been implicated as a potential risk factor for a broad range of medical and psychiatric disorders (Bagby et al., 2020; Taylor et al., 1997, 2024). The 20-item Toronto Alexithymia Scale (TAS-20; Bagby et al., 1994) is the most widely used self-report measure of the construct, comprising three factor-analytically derived subscales: difficulty identifying feelings (DIF), difficulty describing feelings (DDF), and externally oriented thinking (EOT). The TAS-20 has demonstrated good validity and reliability across diverse populations and languages (Bagby et al., 2020; Sekely et al., 2018), and its three-factor structure is highly replicable (Schroeders et al., 2022). Although TAS-20 scores may increase in the context of distressed states, studies across a variety of medical and psychiatric disorders and community samples have demonstrated evidence of relative stability (i.e., relative differences in TAS-20 scores among a sample of individuals remain the same over time even in the context of change in illness symptomatology or levels of distress) (see, Bagby et al., 2020).

During the early 2000s, some investigators evoked uncertainty about the validity of the TAS-20 by reporting research findings suggesting that the scale measures general psychological distress or negative affects rather than alexithymia itself. Leising et al. (2009) characterized the TAS-20 as a measure of general psychological distress on the basis of subscale-level factor analytic evidence in a small clinical and non-clinical sample; and Marchesi et al. (2000) reported some overlap between anxiety and the DIF factor scale of the TAS-20 in a sample comprising patients with anxiety or depressive disorders and patients without any psychiatric disorders. However, several earlier studies, in which other investigators conducted first-order, item-level factor analytic studies with the TAS-20 and different measures of distress (depression, anxiety, somatization), provided evidence that the TAS-20 items load on factors that are clearly separable from distress items that typically formed separate anxiety, depression, and somatization factors (e.g., Bach et al., 1996; Parker et al., 1991). These studies are summarized in a paper by Bagby et al. (2026). More recently, issues regarding whether the TAS-20 distinguishes alexithymia from general psychological distress have re-emerged (see Preece et al., 2020, 2024). Using joint factor analyses of the TAS-20 and measures of depression, anxiety, and stress, Preece and colleagues reported that the TAS-20 DIF subscale cross-loaded substantially on both alexithymia and distress factors, leading these researchers to conclude that the TAS-20 is contaminated by psychological distress and that its DIF subscale may primarily index current distress rather than a stable alexithymic deficit. They have positioned the Perth Alexithymia Questionnaire (PAQ; Preece et al., 2018) as a superior alternative that ostensibly avoids this confound.

These conclusions, however, rest on a narrow evidential base (Preece et al., 2020, 2024). More recently, Bagby et al. (2026) conducted a comprehensive evaluation of the structural discriminant validity of both the TAS-20 and the PAQ in a community sample enriched with participants having psychiatric diagnoses or treatment histories (N = 681). Conducting item-level and then subscale-level exploratory factor analyses with the Depression Anxiety Stress Scales-21 (DASS-21), they found clear separation between alexithymia and distress at both analytic levels, with no meaningful cross-loadings for TAS-20 DIF items on the distress factor. At the subscale level, the TAS-20 DIF subscale loaded with all the PAQ and other TAS-20 subscales on a General Emotional Dysfunction factor with negligible loading on a Psychological Distress factor—a direct ‘non-replication’ of the Preece et al. (2024) finding. These results established that neither the TAS-20 nor the PAQ is structurally compromised by psychological distress.

Notwithstanding these recent outcomes, a critical limitation of the debate as currently framed is its nearly exclusive focus on structural discriminant validity, i.e., whether alexithymia and distress form statistically separable latent factors. Structural evidence addresses the question of whether the two constructs are mathematically distinguishable but leaves unanswered the arguably more important clinical question of whether this distinction matters functionally—that is, whether the variance unique to alexithymia predicts clinically relevant outcomes for which distress measures do not account. To fully establish that the TAS-20 measures something meaningful beyond psychological distress, it must be demonstrated that alexithymia scores predict clinically relevant outcomes incrementally even after the variance shared with distress has been statistically removed. This form of evidence, which we term predictive discriminant validity, provides a direct test of whether the variance unique to alexithymia is functionally and pragmatically consequential rather than merely statistically identifiable (see Bagby et al., 2026; Haynes & Lench, 2003; Hunsley & Meyer, 2003). We use the term predictive discriminant validity in its psychometric sense—referring to the capacity of a measure to predict criterion outcomes incrementally beyond a competing construct (Haynes & Lench, 2003; Hunsley & Meyer, 2003)—rather than in the lay sense of prospective forecasting; concurrent criterion designs are fully recognized in the psychometric literature as providing this form of validity evidence (American Educational Research Association et al., 2014; Messick, 1989). To our knowledge, no study has explicitly examined predictive discriminant validity as a complement to structural analyses in this debate.

Emotion regulation provides a particularly informative criterion domain for evaluating the predictive discriminant validity of alexithymia measures. Theoretically, the core deficits of alexithymia—difficulty identifying and communicating emotional feelings, a concrete, externally oriented cognitive style, and limited imaginal capacity—should constrain the range and effectiveness of emotion regulation strategies available to an individual (Taylor et al., 1997). Effective emotion regulation depends on the ability to recognize, differentiate, and symbolically represent affective states (Gross, 2015; Gratz & Roemer, 2004); individuals with alexithymic traits are compromised in each of these prerequisite capacities. Accordingly, alexithymia should predict emotion dysregulation and use of maladaptive regulation strategies through mechanisms distinct from those by which concurrent distress exerts its effects. While distress may drive maladaptive coping through state-dependent processes such as cognitive narrowing and urgency-driven responding (Tice et al., 2001), alexithymia should contribute through a more stable, trait-based limitation in the cognitive infrastructure required for flexible emotion regulation.

Empirical evidence broadly supports the association between alexithymia and emotion regulation difficulties (Preece et al., 2023). Alexithymia is consistently associated with greater overall emotion dysregulation, greater use of maladaptive cognitive strategies such as rumination and catastrophizing, and greater use of avoidance-oriented behavioral strategies with reduced use of approach-oriented or socially engaged coping. These domains are commonly assessed by the Difficulties in Emotion Regulation Scale (DERS; Gratz & Roemer, 2004), the Cognitive Emotion Regulation Questionnaire (CERQ; Garnefski et al., 2001), and the Behavioral Emotion Regulation Questionnaire (BERQ; Kraaij & Garnefski, 2019), respectively. However, the vast majority of these studies have examined bivariate associations without controlling for psychological distress. Because alexithymia and distress are moderately correlated—correlations that fall within the range expected for a personality vulnerability factor predicting psychopathology (*r* = 0.30 to 0.50; Kotov et al., 2010)—it remains unclear how much of the alexithymia–emotion regulation association reflects unique alexithymic variance versus shared distress variance. This ambiguity is precisely the question that the structural discriminant validity literature has left unresolved.

The present study was designed to address this issue by examining the predictive discriminant validity of the TAS-20 with respect to multiple domains of emotion regulation. Using hierarchical multiple regression, we tested whether the TAS-20—both at the total score and facet levels—predicted global emotion dysregulation (DERS), cognitive emotion regulation strategies (CERQ), and behavioral emotion regulation strategies (Behavioral Emotion Regulation Questionnaire; BERQ; Kraaij & Garnefski, 2019) after controlling for depression, anxiety, and stress as measured by the DASS-21. This study was explicitly designed to extend and complement recent structural discriminant validity findings (Bagby et al., 2026) by shifting the evaluative framework from investigating whether alexithymia and distress are separable constructs to whether the unique variance in alexithymia predicts functionally meaningful outcomes. If the TAS-20 measures a substantive personality trait distinct from psychological distress, its scores should retain significant predictive power over emotion regulation outcomes even when distress is partialed out.

We hypothesized that: (1) the TAS-20 total score would show significant incremental prediction of emotion regulation outcomes beyond psychological distress, providing direct evidence for predictive discriminant validity; and (2) at the facet level, the three TAS-20 subscales would show differential patterns of incremental prediction, with some facets retaining meaningful predictive power in relation to the contribution of distress control, and others showing greater attenuation, reflecting differences in the degree to which each facet captures variance that is distinctively alexithymic versus shared with distress. Specifically, EOT, as a cognitively oriented dimension with no direct analog in distress measures, was expected to retain robust unique predictive power, whereas DIF and DDF, facets more theoretically proximal to undifferentiated emotional arousal than EOT, were expected to show relatively stronger relations to distress, reflecting genuine theoretical overlap rather than measurement artifact.

## 2. Methods

### 2.1. Samples and Participant Recruitment

Participants were a subset (N = 661) of the community sample described in our companion study on structural discriminant validity (Bagby et al., 2026). While the companion study focused on factor analytic structures using the full sample (N = 681), the present study analyzes the specific subset of participants who provided complete data on the criterion emotion regulation measures (DERS, CERQ, BERQ), which were not examined in the previous report. This clinically enriched community sample included 27.8% with a past or current psychiatric diagnosis and 39.9% with a history of treatment, ensuring adequate variance in both alexithymia and distress symptomatology. Participants had a mean age of 46.41 years (*SD* = 16.69) and were 51.30% female, 48.40% male, and 0.20% gender diverse. The sample was ethnically diverse, with 63.20% identifying as White, 13.90% as Black, 8.30% as mixed/bi-racial, 6.80% as Central American, 2.70% as East Asian, and the remainder distributed across other ethnic groups (i.e., Indigenous, Middle/Central Asian, Middle Eastern, South Asian, Southeast Asian). Participants were recruited through the online platform Prolific and completed a battery of questionnaires via Qualtrics. They were required to reside in the U.S. and be able to read English fluently, and they each signed a written consent form. All were paid $7.00 (USD) each if they completed the full battery of questionnaires. Full demographic details are reported in Bagby et al. (2026).

### 2.2. Measures

#### 2.2.1. Toronto Alexithymia Scale-20 (TAS-20)

The TAS-20 (Bagby et al., 1994) is a 20-item self-report questionnaire designed to assess alexithymia according to the original, affect deficit conceptualization of the construct (Nemiah, 1977; Sifneos, 1994; Taylor et al., 2024). Items are rated on a five-point Likert scale, with higher scores reflecting greater alexithymia severity. Although the developers of the scale recommend using only the total score, the instrument comprises three subscales: difficulty identifying feelings (DIF; 7 items), difficulty describing feelings (DDF; 5 items), and externally oriented thinking (EOT; 8 items). The TAS-20 has demonstrated good validity and reliability (Bagby et al., 2020). The EOT subscale’s lower internal consistency reflects the breadth of its item content (see Clark & Watson, 2019) spanning both a pragmatic, externally directed thinking style and a lack of introspection and attention to emotions. This lower reliability likely attenuates the observed regression coefficients and constitutes a limitation that warrants interpretive caution.

#### 2.2.2. Depression Anxiety Stress Scales-21 (DASS-21)

The DASS-21 (Lovibond & Lovibond, 1995) is a 21-item self-report instrument measuring symptoms of depression (DEP), anxiety (ANX), and stress (STR), with seven items per subscale. Items are rated on a four-point Likert scale reflecting experiences over the past week, with higher scores indicating greater symptom severity. Separate subscale scores and a total score representing general psychological distress can be calculated. The DASS-21 demonstrates good to excellent internal consistency across community and clinical samples (Henry & Crawford, 2005) and strong convergent and discriminant validity with related measures of affective symptoms (e.g., BDI-II, HADS). Confirmatory factor analyses typically support the intended three-factor structure, with some studies also identifying a higher-order general distress factor (Lee et al., 2019; Sinclair et al., 2012). It is one of the most widely used screening measures of psychological distress in research and clinical contexts (Antony et al., 1998).

#### 2.2.3. Difficulties in Emotion Regulation Scale (DERS)

The Difficulties in Emotion Regulation Scale (DERS; Gratz & Roemer, 2004) is a 36-item self-report measure designed to assess multiple dimensions of emotion regulation difficulties. Items are rated on a five-point Likert scale ranging from “almost never” to “almost always,” with higher scores reflecting greater emotion dysregulation. The DERS yields a total score and scores for six subscales: Non-acceptance of Emotional Responses, Difficulties Engaging in Goal-Directed Behavior, Impulse Control Difficulties, Lack of Emotional Awareness, Limited Access to Emotion Regulation Strategies, and Lack of Emotional Clarity. The total score is commonly used as a global index of emotion dysregulation. The DERS has demonstrated excellent internal consistency, strong test–retest reliability, and good construct validity across clinical and non-clinical populations (Bardeen et al., 2012; Gratz & Roemer, 2004). Its multidimensional structure has been replicated in numerous samples, and it shows robust associations with psychopathology, affective dysregulation, and maladaptive coping processes (Hallion et al., 2018).

#### 2.2.4. Cognitive Emotion Regulation Questionnaire (CERQ)

The Cognitive Emotion Regulation Questionnaire (CERQ; Garnefski et al., 2001) is a 36-item self-report instrument that assesses cognitive strategies individuals use following stressful or negative events. Items are rated on a five-point Likert scale, with higher scores indicating greater use of the respective cognitive strategy. The CERQ comprises nine subscales: self-blame, acceptance, rumination, positive refocusing, refocus on planning, positive reappraisal, putting into perspective, catastrophizing, and blaming others. These strategies are often grouped into adaptive and maladaptive composites based on theoretical and empirical work, with acceptance, positive refocusing, refocus on planning, positive reappraisal, and putting into perspective considered adaptive, and self-blame, rumination, catastrophizing, and blaming others considered maladaptive (Garnefski et al., 2001; Jermann et al., 2006). The CERQ demonstrates good internal consistency, factorial validity, and convergent validity with measures of emotional distress and coping (Domaradzka & Fajkowska, 2018; Garnefski & Kraaij, 2007). Its factor structure has been replicated across diverse cultural contexts (Miklósi et al., 2011).

#### 2.2.5. Behavioral Emotion Regulation Questionnaire (BERQ)

The Behavioral Emotion Regulation Questionnaire (BERQ; Kraaij & Garnefski, 2019) is a 20-item self-report measure designed to assess behavioral strategies individuals use to regulate emotions following negative or stressful experiences. Items are rated on a five-point Likert scale ranging from “(almost) never” to “(almost) always,” with higher scores reflecting greater use of each strategy. The BERQ comprises five subscales of four items each: seeking distraction, withdrawal, actively approaching, seeking social support, and ignoring. These subscales can be grouped into adaptive (seeking distraction, actively approaching, seeking social support) and maladaptive (withdrawal, ignoring) composites, reflecting approach-oriented and avoidance-oriented behavioral regulation strategies, respectively (Kraaij & Garnefski, 2019); in the present study, we created and used these composite scores as criterion variables. The BERQ demonstrates good internal consistency, a stable five-factor structure, and meaningful associations with emotional problems and cognitive emotion regulation strategies (Kraaij & Garnefski, 2019).

### 2.3. Data Analysis

To examine cognitive and behavioral emotion regulation processes beyond global emotion dysregulation, composite indices of adaptive and maladaptive emotion regulation were created for the CERQ and the BERQ based on the groupings of the subscales outlined above. The CERQ adaptive composite comprised acceptance, positive refocusing, refocus on planning, positive reappraisal, and putting into perspective; the CERQ maladaptive composite comprised self-blame, rumination, catastrophizing, and blaming others (Garnefski et al., 2001; Jermann et al., 2006; Domaradzka & Fajkowska, 2018). The BERQ adaptive composite comprised seeking distraction, actively approaching, and seeking social support; the BERQ maladaptive composite comprised withdrawal and ignoring (Kraaij & Garnefski, 2019). All composites were computed as the sum of the constituent subscale scores; higher composite scores indicated greater reported use of the respective adaptive or maladaptive regulation strategies. In the present sample, internal consistency for the CERQ adaptive composite was α = 0.91, and α = 0.87 for the CERQ maladaptive composite; internal consistency for the BERQ adaptive composite was α = 0.86, and α = 0.90 for the BERQ maladaptive composite.

To investigate the predictive discriminant validity of the TAS-20, a series of two-step hierarchical multiple regression analyses was conducted. This analytic approach directly addresses ongoing concerns regarding the construct validity of the TAS-20 and its overlap with general psychological distress (Preece et al., 2020, 2024) by disentangling shared from unique variance in predicting emotion regulation outcomes.

To this end, we utilized a hierarchical entry order in which the DASS-21 distress subscales (depression, anxiety, and stress) were entered as a block in Step 1, followed by the TAS-20 predictors (total score or facet scale scores) in Step 2. This entry order follows standard psychometric procedures for evaluating incremental validity (Haynes & Lench, 2003; Hunsley & Meyer, 2003), in which the competing construct is partialed first so that the change in *R*^2^ at Step 2 directly indexes the unique contribution of the focal measure (TAS-20) beyond what general distress already explains. Two separate sets of models were conducted: the first set entered the TAS-20 total score in Step 2, and the second set entered the three TAS-20 subscales (DIF, DDF, EOT) simultaneously in Step 2. For reference, Hunsley and Meyer (2003) proposed a Δ*R*^2^ of 0.15 as a heuristic benchmark for clinically meaningful incremental validity; we report the Δ*R*^2^ as the primary index of the TAS-20’s incremental contribution and additionally report the squared semi-partial correlation (*sr*^2^) from the full model as a complementary, entry-order-invariant metric for comparing the unique contributions of alexithymia and the individual distress subscales within the same model. Power analyses were conducted using the *F*-test for *R*^2^ increment (linear multiple regression, fixed model) with alpha = 0.05. With analytic *N*s ranging from 602 to 640, the minimum detectable Δ*R*^2^ at 80% power ranged from 0.004 to 0.012 for total-score models (*k*_added = 1, *k*_total = 4) and from 0.005 to 0.017 for subscale models (*k*_added = 3, *k*_total = 6). Achieved power for all observed increments exceeded 0.89 and reached 1.00 for three of five total-score outcomes. These analyses confirm that the present sample is more than adequately powered to detect even small incremental effects, and that statistical significance alone is not a sufficient interpretive threshold.

To compare the relatively unique contributions of individual predictors within the final (Step 2) models, we additionally report squared semi-partial correlations (*sr*^2^) derived from the standardized regression coefficients. The squared semi-partial correlation indexes the proportion of total variance in the criterion that is uniquely attributable to a given predictor after removing variance shared with all other predictors in the model; *sr*^2^ values computed from the full model are invariant to entry order, making them an appropriate metric for comparing the unique explanatory contributions of alexithymia and individual distress subscales within the same model. The Δ*R*^2^ at Step 2 directly quantifies the unique contribution of the TAS-20 beyond depression, anxiety, and stress; the *sr*^2^ values complement this by allowing predictor-by-predictor comparisons of unique contributions within the final model.

Finally, because different emotion regulation measures were completed by slightly different subsets of participants, sample sizes varied across regression models due to listwise deletion. Thus, *N*s and associated degrees of freedom differ modestly across outcomes (e.g., DERS, CERQ, BERQ models). The full sample comprised N = 661 participants, but analyses for each outcome included only those participants with complete data for all variables entered in the respective regression model; however, the differences in sample sizes across the various analyses were minimal.

## 3. Results

### 3.1. Preliminary Analyses

Descriptive statistics for all study variables are presented in Table 1. Across measures, mean scores and observed ranges were consistent with those reported in prior non-clinical and mixed samples, and no evidence of severe departures from normality was observed. Skewness and kurtosis values for all variables fell within acceptable limits, indicating that assumptions of approximate normality were met for subsequent parametric analyses. Internal consistency estimates were in general uniformly strong. The DERS total and subscale scores demonstrated excellent reliability. The TAS-20 total score and its DIF and DDF subscales also showed good to excellent internal consistency; however, the internal reliability for EOT was comparatively lower (alpha = 0.64). While this falls below the conventional 0.70 threshold, it is consistent with the prior literature and reflects the broad conceptualization of this facet of the alexithymia construct that we noted earlier (Bagby et al., 2020; Müller et al., 2003); lower reliability also increases error variance in regression analyses, and interpretations of EOT coefficients should be made with appropriate caution. The DASS-21 subscales demonstrated strong internal consistency across depression, anxiety, and stress. For emotion regulation composites, both CERQ and BERQ adaptive and maladaptive indices exhibited good to excellent reliability, supporting their use in subsequent regression analyses. As noted in the Data Analysis section, sample sizes varied modestly across regression models due to listwise deletion (ranging from N = 602 to N = 640); comparison of descriptive statistics across these subsamples indicated no meaningful differences in demographic composition or predictor distributions, confirming that the fluctuations did not systematically bias the analytic samples.

### 3.2. Bivariate Correlations

Zero-order correlations among measures of emotion dysregulation, alexithymia, psychological distress, and adaptive and maladaptive emotion regulation composites are presented in Supplementary Table S1. As expected, global emotion dysregulation (DERS total) was strongly and positively correlated with TAS-20 total scores (*r* = 0.72), reflecting a large effect. Similarly large positive associations were observed with DIF (*r* = 0.72) and DDF (*r* = 0.68), whereas the association with EOT was moderate in magnitude (*r* = 0.32). DERS total also showed large positive correlations with overall psychological distress (DASS-21 total; *r* = 0.77), stress (*r* = 0.72), and depression (*r* = 0.74), and a moderate-to-large association with anxiety (*r* = 0.59). These findings indicate substantial shared variance among alexithymia, distress, and global emotion dysregulation.

At the facet level, DIF and DDF were very strongly intercorrelated (*r* = 0.77; large effect), consistent with their shared content of affect identification and articulation. Both were strongly associated with overall distress (DIF with DASS total, *r* = 0.63; DDF with DASS total, *r* = 0.55; large effects). In contrast, EOT demonstrated only a small association with distress (*r* = 0.17), supporting its relative distinctiveness from general psychological distress at the zero-order level.

For behavioral emotion regulation, the BERQ maladaptive composite showed large positive correlations with TAS-20 total (*r* = 0.60), DIF (*r* = 0.55), and DDF (*r* = 0.61), and a moderate association with EOT (*r* = 0.28). Associations with psychological distress were also large (DASS total, *r* = 0.61; depression, *r* = 0.63; stress, *r* = 0.55) and moderate for anxiety (*r* = 0.45). Thus, maladaptive behavioral regulation was robustly associated with both alexithymia and distress. In contrast, the BERQ adaptive composite demonstrated moderate negative correlations with TAS-20 total (*r* = −0.35), DDF (*r* = −0.34), and EOT (*r* = −0.31), and a small-to-moderate negative association with DIF (*r* = −0.24). Associations with distress were small to moderate (DASS total, *r* = −0.18; depression, *r* = −0.27; stress, *r* = −0.11), and negligible for anxiety (*r* = −0.05). This pattern suggests that adaptive behavioral regulation is more strongly and consistently linked (negatively) to alexithymic traits than to concurrent psychological distress.

For cognitive emotion regulation, the CERQ maladaptive composite showed a moderate positive correlation with TAS-20 total (*r* = 0.30), and moderate associations with DIF (*r* = 0.40) and DDF (*r* = 0.33), whereas EOT was essentially unrelated at the zero-order level (*r* = −0.04). In contrast, distress demonstrated large associations with maladaptive cognitive regulation (DASS total, *r* = 0.60; stress, *r* = 0.59; anxiety, *r* = 0.51; depression, *r* = 0.51). This pattern indicates that maladaptive cognitive strategies are more strongly tied to current distress than to alexithymic traits per se. The CERQ adaptive composite was modestly negatively correlated with TAS-20 total (*r* = −0.19), DIF (*r* = −0.14), DDF (*r* = −0.15), and EOT (*r* = −0.18), all with small effects. Associations with distress were similarly small (DASS total, *r* = −0.14; depression, *r* = −0.21; stress, *r* = −0.10; anxiety, *r* = −0.02), indicating comparatively weaker and more differentiated relationships in the adaptive cognitive domain.

Importantly, although alexithymia and distress were moderately to strongly intercorrelated (TAS-20 total with DASS total, *r* = 0.58; large effect), none of the correlations approached levels typically indicative of problematic multicollinearity (e.g., *r* ≥ 0.80), supporting the inclusion of TAS-20 and DASS-21 variables as simultaneous predictors in the hierarchical regression models.

### 3.3. Hierarchical Regression Results Using TAS-20 Total Score

A series of two-step hierarchical multiple regression analyses was conducted to examine whether the TAS-20 total score predicted emotion dysregulation and emotion regulation strategy use after accounting for psychological distress. Preliminary diagnostics indicated that all models satisfied assumptions of linearity, normality of residuals, homoscedasticity, and independence of errors. No influential outliers were detected, and multicollinearity diagnostics were within acceptable limits (VIF < 5; tolerance > 0.20 across all models).

#### 3.3.1. DERS Total Score

As displayed in Table 2, when predicting global emotion dysregulation (DERS total), Step 1 was significant, *F*(3, 598) = 329.39, *p* < 0.001, *R*^2^ = 0.62. Stress (*β* = 0.39, *p* < 0.001) and depression (*β* = 0.45, *p* < 0.001) were significant predictors, whereas anxiety was not (*β* = 0.01, *p* = 0.71). After adding the TAS-20 total score in Step 2, the model remained significant, *F*(4, 597) = 390.26, *p* < 0.001, *R*^2^ = 0.72, reflecting a significant increase in explained variance (Δ*R*^2^ = 0.10, *F*(1, 597) = 213.21, *p* < 0.001).

In the final model, the TAS-20 total score was a robust unique predictor (*β* = 0.39, *p* < 0.001), accounting for approximately 10% of the variance in DERS total beyond depression, anxiety, and stress (*sr*^2^ = 0.10). Depression (*β* = 0.32, *p* < 0.001; *sr*^2^ = 0.04) and stress (*β* = 0.32, *p* < 0.001; *sr*^2^ = 0.04) retained unique contributions in the final model, whereas anxiety did not (*β* = −0.02, *p* = 0.46; *sr*^2^ ≈ 0.00). These findings indicate that the TAS-20 accounts for approximately 10 percentage points of unique variance in global emotion dysregulation beyond concurrent depression, anxiety, and stress, and that within the final model the TAS-20 total score uniquely accounts for a larger share of variance than any individual distress subscale.

#### 3.3.2. BERQ Composite Scales

For adaptive behavioral emotion regulation (Table 3), Step 1 was significant, *F*(3, 620) = 23.15, *p* < 0.001, *R*^2^ = 0.10. Depression was the strongest predictor (*β* = −0.43, *p* < 0.001) and anxiety was also significant (*β* = 0.19, *p* < 0.001), whereas stress was not (*β* = 0.05, *p* = 0.41). After adding the TAS-20 total score in Step 2, the model remained significant, *F*(4, 619) = 34.17, *p* < 0.001, *R*^2^ = 0.18, with a significant increase in explained variance (Δ*R*^2^ = 0.08, *F*(1, 619) = 60.42, *p* < 0.001). In the final model, the TAS-20 total score was a robust unique predictor (*β* = −0.35, *p* < 0.001), uniquely accounting for approximately 8% of the variance in adaptive behavioral regulation beyond distress (*sr*^2^ = 0.08). Depression (*β* = −0.32, *p* < 0.001; *sr*^2^ = 0.04), anxiety (*β* = 0.22, *p* < 0.001; *sr*^2^ = 0.02), and stress (*β* = 0.13, *p* < 0.05; *sr*^2^ = 0.01) retained smaller unique contributions in the final model. The TAS-20, thus, accounted for a larger share of unique variance in adaptive behavioral regulation than any individual distress subscale.

For maladaptive behavioral emotion regulation, Step 1 was significant, *F*(3, 626) = 144.03, *p* < 0.001, *R*^2^ = 0.41, with depression (*β* = 0.48, *p* < 0.001) and stress (*β* = 0.22, *p* < 0.001) as significant predictors and anxiety non-significant (*β* = −0.02, *p* = 0.67). After adding the TAS-20 total score in Step 2, the model remained significant, *F*(4, 625) = 144.52, *p* < 0.001, *R*^2^ = 0.48 (Δ*R*^2^ = 0.07, *F*(1, 625) = 84.13, *p* < 0.001). In the final model, the TAS-20 total score was a significant unique predictor (*β* = 0.33, *p* < 0.001; *sr*^2^ = 0.07). Depression (*β* = 0.37, *p* < 0.001; *sr*^2^ = 0.06) and stress (*β* = 0.15, *p* < 0.01; *sr*^2^ = 0.01) also contributed significantly, whereas anxiety did not (*β* = −0.05, *p* = 0.25). Alexithymia retained meaningful unique prediction of maladaptive behavioral strategies after controlling for depression, anxiety, and stress, accounting for a larger share of unique variance than any individual distress subscale.

#### 3.3.3. CERQ Composite Scales

For adaptive cognitive emotion regulation (Table 4), Step 1 was significant, *F*(3, 610) = 15.80, *p* < 0.001, *R*^2^ = 0.07, with anxiety (*β* = 0.23, *p* < 0.001) and depression (*β* = −0.34, *p* < 0.001) as significant predictors and stress non-significant (*β* = −0.03, *p* = 0.61). After adding the TAS-20 total score in Step 2, the model remained significant, *F*(4, 609) = 14.54, *p* < 0.001, *R*^2^ = 0.09 (Δ*R*^2^ = 0.02, *F*(1, 609) = 13.39, *p* < 0.001). In the final model, the TAS-20 total score was a significant unique predictor (*β* = −0.15, *p* < 0.001), uniquely accounting for approximately 2% of the variance in adaptive cognitive regulation beyond distress (*sr*^2^ = 0.02). Depression (*β* = −0.29, *p* < 0.001; *sr*^2^ = 0.04) and anxiety (*β* = 0.24, *p* < 0.001; *sr*^2^ = 0.03) accounted for larger unique portions in the final model, whereas stress did not contribute (*β* = 0.00, *p* = 0.98). The TAS-20 retained statistically significant incremental prediction of adaptive cognitive regulation, although in this domain distress accounted for a larger share of unique variance than alexithymia.

For maladaptive cognitive emotion regulation, Step 1 was significant, *F*(3, 620) = 121.53, *p* < 0.001, *R*^2^ = 0.37, with stress (*β* = 0.38, *p* < 0.001), anxiety (*β* = 0.14, *p* = 0.003), and depression (*β* = 0.14, *p* = 0.002) being all significant. After adding the TAS-20 total score in Step 2, the model remained significant, *F*(4, 619) = 92.88, *p* < 0.001, *R*^2^ = 0.38 (Δ*R*^2^ = 0.01, *F*(1, 619) = 9.98, *p* = 0.002). In the final model, the TAS-20 total score showed a small negative association (*β* = −0.08, *p* < 0.05; *sr*^2^ = 0.01), indicating that once distress was partialed out, the relationship between alexithymia and maladaptive cognitive regulation exhibited reversed sign. Stress emerged as the strongest predictor in the final model (*β* = 0.40, *p* < 0.001; *sr*^2^ = 0.06), followed by depression (*β* = 0.17, *p* < 0.001; *sr*^2^ = 0.01) and anxiety (*β* = 0.15, *p* < 0.01; *sr*^2^ = 0.01). These findings indicate that maladaptive cognitive strategies are primarily associated with concurrent psychological distress; the TAS-20 contributed only a small significant increment beyond distress, and the negative sign of its coefficient in the final model is consistent with a suppression effect.

### 3.4. Hierarchical Regression Results Using TAS-20 Facet-Scales

A second series of two-step hierarchical multiple regression analyses was conducted to examine whether specific facets of alexithymia predicted emotion dysregulation and the use of emotion regulation strategies after accounting for psychological distress. As in the total-score models, diagnostics indicated that assumptions of linearity, normality of residuals, and homoscedasticity were satisfied. No influential cases were detected, and multicollinearity indices were within acceptable limits (VIF < 5; tolerance > 0.20 across all models). Because of the substantial intercorrelation among the TAS-20 subscales (particularly between DIF and DDF, *r* = 0.77), the *sr*^2^ values for individual facets in these models represent their unique contributions after controlling for the other facets as well as distress and, thus, constitute a conservative test of each facet’s predictive power.

#### 3.4.1. DERS Total Score

As shown in Table 5, when predicting DERS total, Step 1 was significant, *F*(3, 598) = 329.39, *p* < 0.001, *R*^2^ = 0.62. Stress (*β* = 0.39, *p* < 0.001) and depression (*β* = 0.45, *p* < 0.001) were significant predictors, whereas anxiety was not (*β* = 0.01, *p* = 0.71). Squared semi-partial correlations indicated that depression uniquely accounted for approximately 10% of the variance (*sr*^2^ = 0.10) and stress uniquely accounted for approximately 6% (*sr*^2^ = 0.06), whereas anxiety contributed negligibly.

Step 2, in which the three TAS-20 subscales were added simultaneously, remained significant, *F*(6, 595) = 266.14, *p* < 0.001, *R*^2^ = 0.73, reflecting a significant increase in explained variance (Δ*R*^2^ = 0.11, *F*(3, 595) = 80.78, *p* < 0.001). In the final model, DIF (*β* = 0.23, *p* < 0.001), DDF (*β* = 0.18, *p* < 0.001), and EOT (*β* = 0.06, *p* < 0.05) were each significant unique predictors after controlling for distress. Squared semi-partial correlations indicated that DIF uniquely accounted for approximately 2% of the variance beyond distress (*sr*^2^ = 0.02), DDF approximately 1% (*sr*^2^ = 0.01), and EOT contributed negligibly (*sr*^2^ ≈ 0.00). Distress predictors retained their contributions in the final model, with depression accounting for approximately 4% of unique variance (*sr*^2^ = 0.04) and stress approximately 3% (*sr*^2^ = 0.03), whereas anxiety contributed minimally. Although the alexithymia subscale coefficients are constrained by their substantial intercorrelation (particularly DIF and DDF, *r* = 0.77), DIF, DDF, and EOT each retained statistically significant incremental prediction of global emotion dysregulation beyond depression, anxiety, and stress.

#### 3.4.2. BERQ Composite Scales

For BERQ adaptive (Table 6), Step 1 was significant, *F*(3, 620) = 23.15, *p* < 0.001, *R*^2^ = 0.10, with depression (*β* = −0.43, *p* < 0.001) and anxiety (*β* = 0.19, *p* < 0.001) as significant predictors and stress non-significant (*β* = 0.05, *p* = 0.41). Step 2, in which the three TAS-20 subscales were added simultaneously, remained significant, *F*(6, 617) = 25.04, *p* < 0.001, *R*^2^ = 0.20, with a significant increase in explained variance (Δ*R*^2^ = 0.10, *F*(3, 617) = 25.71, *p* < 0.001). In the final model, DDF (*β* = −0.25, *p* < 0.001) and EOT (*β* = −0.17, *p* < 0.001) emerged as significant unique alexithymia predictors after controlling for distress, whereas DIF did not (*β* = 0.02, *p* = 0.80). Squared semi-partial correlations indicated that DDF uniquely accounted for approximately 2% of the variance beyond distress (*sr*^2^ = 0.02) and EOT for another approximate 2% (*sr*^2^ = 0.02). Among distress predictors in the final model, depression accounted for approximately 4% (*sr*^2^ = 0.04) and anxiety for approximately 2% (*sr*^2^ = 0.02), whereas stress did not reach conventional significance (*β* = 0.11, *p* = 0.06). DDF and EOT, therefore, retained incremental predictive value for reduced adaptive behavioral regulation after depression, anxiety, and stress were partialed out.

For BERQ maladaptive, Step 1 was significant, *F*(3, 626) = 144.03, *p* < 0.001, *R*^2^ = 0.41, with depression (*β* = 0.48, *p* < 0.001) and stress (*β* = 0.22, *p* < 0.001) as significant predictors and anxiety non-significant (*β* = −0.02, *p* = 0.67). Step 2, in which the three TAS-20 subscales were added simultaneously, remained significant, *F*(6, 623) = 102.84, *p* < 0.001, *R*^2^ = 0.50, with a significant increase in explained variance (Δ*R*^2^ = 0.09, *F*(3, 623) = 37.40, *p* < 0.001). In the final model, DDF was the only significant alexithymia predictor (*β* = 0.33, *p* < 0.001; *sr*^2^ = 0.04); DIF (*β* = −0.01, *p* = 0.88) and EOT (*β* = 0.05, *p* = 0.10) did not reach significance after distress was controlled. Among distress predictors, depression accounted for approximately 5% of unique variance (*sr*^2^ = 0.05) and stress accounted for approximately 1% (*sr*^2^ = 0.01), whereas anxiety did not contribute. DDF, therefore, retained a robust and statistically significant unique association with maladaptive behavioral regulation after controlling for depression, anxiety, and stress.

#### 3.4.3. CERQ Composite Scales

For CERQ adaptive (Table 7), Step 1 was significant, *F*(3, 610) = 15.80, *p* < 0.001, *R*^2^ = 0.07, with anxiety (*β* = 0.23, *p* < 0.001) and depression (*β* = −0.34, *p* < 0.001) as significant predictors and stress non-significant (*β* = −0.03, *p* = 0.61). Step 2, in which the three TAS-20 subscales were added simultaneously, remained significant, *F*(6, 607) = 10.11, *p* < 0.001, *R*^2^ = 0.09, with a significant increase in explained variance (Δ*R*^2^ = 0.02, *F*(3, 607) = 4.45, *p* = 0.004). In the final model, EOT was the only significant alexithymia predictor (*β* = −0.11, *p* = 0.009; *sr*^2^ = 0.01); DIF (*β* = −0.06, *p* = 0.36) and DDF (*β* = −0.01, *p* = 0.88) did not contribute. Among distress predictors, depression accounted for approximately 4% of unique variance (*sr*^2^ = 0.04) and anxiety accounted for approximately 2% (*sr*^2^ = 0.02), whereas stress did not contribute. EOT, therefore, retained a small but statistically significant unique association with reduced adaptive cognitive regulation after controlling for depression, anxiety, and stress, although in this domain distress accounted for the larger share of unique variance.

For CERQ maladaptive, Step 1 was significant, *F*(3, 620) = 121.53, *p* < 0.001, *R*^2^ = 0.37, with stress (*β* = 0.38, *p* < 0.001), anxiety (*β* = 0.14, *p* = 0.003), and depression (*β* = 0.14, *p* = 0.002) being all significant. Step 2, in which the three TAS-20 subscales were added simultaneously, remained significant, *F*(6, 617) = 67.65, *p* < 0.001, *R*^2^ = 0.40, with a significant increase in explained variance (Δ*R*^2^ = 0.03, *F*(3, 617) = 10.28, *p* < 0.001). In the final model, only EOT remained a significant alexithymia predictor (*β* = −0.18, *p* < 0.001; *sr*^2^ = 0.03); DIF (*β* = 0.04, *p* = 0.46) and DDF (*β* = 0.04, *p* = 0.48) did not contribute after distress was controlled. Stress emerged as the strongest predictor in the final model (*β* = 0.37, *p* < 0.001; *sr*^2^ = 0.05), followed by depression (*β* = 0.17, *p* < 0.001; *sr*^2^ = 0.01) and anxiety (*β* = 0.12, *p* < 0.05; *sr*^2^ = 0.01). The negative association for EOT was more pronounced in the final model than at the zero-order level, which is consistent with a statistical suppression effect. EOT, therefore, uniquely predicts lower engagement in maladaptive cognitive elaboration strategies beyond depression, anxiety, and stress.

## 4. Discussion

### 4.1. Primary Findings

The present study examines the predictive discriminant validity of the TAS-20 in the context of the ongoing debate about whether this instrument adequately distinguishes alexithymia from psychological distress. While prior investigations have focused almost exclusively on structural discriminant validity, examining whether alexithymia and distress form separable latent factors in factor analytic studies (Bagby et al., 2026; Preece et al., 2020, 2024), the present study addressed a complementary and arguably more consequential question: does the variance captured by the TAS-20 predict functionally meaningful outcomes beyond what psychological distress alone accounts for? Across multiple domains of emotion regulation, the TAS-20 retained statistically significant incremental prediction, although the magnitude of incremental prediction varied meaningfully across outcome domains. At the total-score level, the TAS-20 retained statistically significant incremental prediction across all five emotion regulation outcomes after controlling for depression, anxiety, and stress. The strongest increments were observed for global emotion dysregulation and behavioral emotion regulation, whereas the increments for cognitive emotion regulation outcomes were smaller. Thus, the findings support the predictive discriminant validity of the TAS-20, while also indicating that its incremental contribution is domain-specific rather than uniform across all forms of emotion regulation. These findings should be interpreted as quantifying, rather than dissolving, the discriminant validity question concerning alexithymia and distress: the increments are real, theoretically interpretable, and statistically reliable, but they are also modest, and they confirm meaningful overlap between the two constructs.

The strongest total-score evidence emerged for global emotion dysregulation and behavioral emotion regulation. For DERS total, the TAS-20 total score added a statistically significant increment beyond depression, anxiety, and stress (Δ*R*^2^ = 0.10) and accounted for a larger unique share of variance than any individual distress subscale in the final model. For the BERQ outcomes, the TAS-20 also retained meaningful incremental prediction beyond distress, adding Δ*R*^2^ = 0.08 for adaptive behavioral regulation and Δ*R*^2^ = 0.07 for maladaptive behavioral regulation. By contrast, the TAS-20 increments for the CERQ outcomes were smaller (Δ*R*^2^ = 0.02 for adaptive cognitive regulation and Δ*R*^2^ = 0.01 for maladaptive cognitive regulation), suggesting that cognitive emotion regulation strategies are more closely tied to current distress than behavioral regulation outcomes. Thus, the evidence for predictive discriminant validity was statistically reliable across domains but strongest for global and behavioral regulation outcomes.

It should be acknowledged that two DERS subscales in particular—Lack of Emotional Awareness and Lack of Emotional Clarity—share clear conceptual ground with the DIF and DDF facets of alexithymia, respectively, given that difficulty identifying and describing feelings overlaps substantially in content with impaired awareness and clarity of emotional states. Associations between the TAS-20 and these specific DERS subscales may, therefore, partly reflect shared item content in addition to external criterion prediction, and the DERS total score findings should be interpreted with this caveat in mind. To the extent that shared item content inflates the TAS-20–DERS association, the incremental *sr*^2^ values reported for global emotion dysregulation should be regarded as upper-bound estimates of the TAS-20’s unique predictive contribution; the CERQ and BERQ findings, which are free of this concern, provide more conservative evidence.

It should be acknowledged that the obtained Δ*R*^2^ values fall below the 0.15 heuristic proposed by Hunsley and Meyer (2003) for clinically meaningful incremental validity. Across the five total-score outcomes, increments ranged from 0.01 to 0.10 (median = 0.07); the corresponding subscale-level increments ranged from 0.02 to 0.11 (median = 0.09). By the strictest application of this guideline, the TAS-20’s incremental contribution beyond DASS-21 distress would be characterized as small for global emotion dysregulation and behavioral regulation outcomes and as small-to-negligible for cognitive regulation outcomes. We do not regard this as a trivial observation. The accumulated structural and predictive evidence indicates that alexithymia and distress are distinct but substantially overlapping constructs, and the present results quantify the cost of that overlap for the TAS-20’s incremental utility.

Several considerations bear on interpretation. First, the lower internal consistency of the EOT subscale (α = 0.64) attenuates its regression coefficients and the Δ*R*^2^ to which it contributes, and EOT estimates should accordingly be regarded as conservative. Second, the present design imposes a deliberately stringent test: alexithymia is theoretically and empirically correlated with general distress in the *r* = 0.30–0.50 range (Bagby et al., 2020; Kotov et al., 2010), so partialing distress in Step 1 removes substantial construct-relevant variance before the TAS-20 is given any opportunity to predict. Third, Hunsley and Meyer’s heuristic was articulated for evaluating a candidate measure against a single, narrowly overlapping competitor; its application is less straightforward when the competitor is operationalized through three correlated subscales that share moderate trait-level variance with the focal construct.

Situated within the broader literature, incremental contributions in the 0.07–0.10 range are consistent with what is typically observed when a personality trait measure is required to predict outcomes beyond a comprehensive measure of concurrent affective symptomatology; comparably modest incremental effects are routinely observed when constructs from the internalizing spectrum are evaluated against one another (Kotov et al., 2010). The present pattern—statistically reliable, theoretically coherent, but modest in magnitude—is best interpreted as evidence that the TAS-20 captures incremental variance that is meaningful but not large. The cognitive regulation findings, where increments dropped to 0.02–0.03, mark the limit of what the TAS-20 adds beyond distress in this sample; the global and behavioral findings, where increments approached but did not reach the 0.15 benchmark, are the strongest evidence of incremental value. Neither outcome supports the strong claim that the TAS-20 is reducible to distress, nor does either outcome support an unqualified claim of robust independence; both characterize alexithymia and distress as related but separable contributors to emotion regulation, with the TAS-20’s added value most apparent in domains less saturated with affective symptom content.

These findings directly address the central concern raised by Preece et al. (2020, 2024). If the TAS-20 primarily measured psychological distress rather than a distinct alexithymia construct, its predictive power should collapse once distress is controlled. This was not the case here; the TAS-20 demonstrated statistically significant, domain-differentiated incremental prediction across the emotion regulation outcomes, indicating that variance captured by the TAS-20 beyond distress is functionally consequential for how individuals regulate their emotions.

### 4.2. Differential Facet-Level Patterns

The facet-level analyses revealed a richly differentiated pattern that is both psychometrically informative and theoretically coherent. Three distinct profiles emerged across the DIF, DDF, and EOT subscales, each illuminating different aspects of the alexithymia–distress–emotion regulation nexus. It should be noted that although individual alexithymia facets—particularly DDF and EOT—emerged as robust predictors, the combined unique variance of the three TAS-20 subscales was smaller than the combined distress contribution across all facet-level models. This reflects, in part, some multicollinearity among the alexithymia components, especially DIF and DDF, compared to the distress subscales, which reduces the unique contribution when all three TAS-20 subscales are entered simultaneously. The facet-level analyses are, therefore, best understood as revealing which components of alexithymia carry the unique predictive signal, rather than as a direct comparison of the magnitude of alexithymia versus distress contributions.

#### 4.2.1. Difficulty Describing Feelings (DDF)

DDF emerged as the most consistent unique alexithymia predictor across emotion regulation outcomes after controlling for distress. It retained significant incremental prediction of global emotion dysregulation (DERS; *β* = 0.18), reduced adaptive behavioral regulation (BERQ adaptive; *β* = −0.25), and increased maladaptive behavioral regulation (BERQ maladaptive; *β* = 0.33). Although DDF was predicted to show relatively stronger distress-related attenuation, given its theoretical proximity to affective disturbance, unique predictive power proved more robust than anticipated, a departure from the directional prediction that itself carries theoretical significance. For maladaptive behavioral regulation, DDF remained a significant unique predictor after distress was controlled (*β* = 0.33; *sr*^2^ = 0.04), with depression accounting for a somewhat larger share of unique variance (*sr*^2^ = 0.05). Although the bivariate DDF–maladaptive behavioral regulation association was substantially reduced by distress control, the residual unique contribution of DDF—approximately four percentage points of variance—remained statistically reliable and theoretically interpretable, reflecting a stable, trait-based deficit in emotional articulation that is not reducible to concurrent distress.

This pattern is theoretically coherent. Difficulty describing feelings represents a deficit in the symbolic articulation of emotional experience—an impairment in verbally communicating felt affect to others. This capacity is a prerequisite for socially engaged regulation strategies (e.g., seeking social support, actively approaching problems), and its absence would logically drive individuals toward avoidance-oriented behavioral responses such as withdrawal and emotional ignoring, which are the strategies comprising the BERQ maladaptive composite. The robustness of DDF after distress control underscores that this is a stable, trait-like deficit in emotional communication that operates independently of how distressed an individual currently feels.

#### 4.2.2. Externally Oriented Thinking (EOT)

EOT displayed a distinctive pattern of incremental prediction concentrated in the strategy domains rather than global dysregulation. While its contribution to DERS total was modest (*β* = 0.06, *p* < 0.05), it retained significant predictive power for both adaptive cognitive regulation (CERQ adaptive; *β* = −0.11) and maladaptive cognitive regulation (CERQ maladaptive; *β* = −0.18) after controlling for distress. It also retained prediction of reduced adaptive behavioral regulation (BERQ adaptive; *β* = −0.17). The direction of the EOT–CERQ maladaptive association merits attention: higher externally oriented thinking predicted lower use of maladaptive cognitive strategies such as rumination, catastrophizing, self-blame, and blaming others. This negative association is theoretically consistent with the nature of externally oriented thinking as a concrete, pragmatic, stimulus-bound cognitive style characterized by reduced attention to emotional and other internal states (Taylor et al., 1997, 2023). Maladaptive cognitive regulation strategies such as rumination and catastrophizing require sustained introspective engagement with emotional content, precisely the kind of internally directed cognitive activity that individuals high in EOT tend not to engage in. EOT may, thus, represent a form of cognitive inflexibility that limits the entire repertoire of deliberate cognitive regulation, whether adaptive or maladaptive, rather than selectively impairing one type.

This interpretation aligns with and extends the findings from the companion structural discriminant validity study (Bagby et al., 2026), in which EOT items from both the TAS-20 and the Perth Alexithymia Questionnaire loaded negatively on the Psychological Distress Factor at the item level. Together, these structural and predictive findings converge on the conclusion that externally oriented thinking represents a facet of alexithymia that is not only structurally distinct from distress but also functionally distinct in how it relates to emotion regulation processes. The fact that EOT has no direct analog in distress measures makes it a particularly informative marker of unique alexithymic variance. It should also be noted that EOT’s internal consistency in the present sample (α = 0.64, ω = 0.63) was lower than that of DIF and DDF, consistent with its broad item content assessing both a pragmatic thinking style and limited introspection (Clark & Watson, 2019). This lower reliability likely attenuates the observed regression coefficients of EOT, suggesting that the incremental predictive effects reported here may underestimate its true contribution to emotion regulation outcomes.

#### 4.2.3. Difficulty Identifying Feelings (DIF)

DIF exhibited some reductions across various outcomes concerning psychological distress. Although it maintained a notable predictive capacity of overall emotion dysregulation (DERS; *β* = 0.23), its distinct contributions to behavioral and cognitive emotion regulation outcomes were in some instances (i.e., maladaptive BERQ and CERQ) attenuated to non-significance once distress was included in the model. This suggests that DIF shares more variance with psychological distress compared to DDF or EOT, aligning with the subscale-level cross-loading patterns observed by Preece et al. (2020, 2024).

However, the interpretation of DIF attenuation requires careful consideration. Greater shared variance between DIF and distress does not necessarily indicate that DIF is measuring distress rather than alexithymia. The theoretical framework of alexithymia explicitly positions difficulty identifying feelings as the facet most proximally related to affective disturbance: individuals who cannot identify what they are feeling are, by definition, more likely to experience undifferentiated emotional arousal that manifests as anxiety, depression, and stress (Sifneos, 1967; Taylor et al., 1997). The overlap between DIF and distress may, therefore, reflect measurement overlap, a genuine causal pathway (alexithymic deficits in emotion identification, increasing distress), or a combination of both—our cross-sectional data cannot distinguish among these possibilities. Importantly, if a causal pathway is operative, as the theoretical framework of alexithymia would predict, then controlling for distress in the regression models would partial out not only nuisance variance but also some of the very mechanisms through which DIF contributes to emotion dysregulation, meaning the resulting attenuation would underestimate the true functional importance of DIF. This possibility should temper interpretations that treat DIF attenuation as evidence of measurement contamination. Neuroimaging studies suggest that the opposite effect of identifying and labeling emotional feelings can alleviate emotional distress by increasing activity in the right ventrolateral prefrontal cortex, which, in turn, dampens activity in the amygdala (Lieberman et al., 2007, 2011).

Critically, DIF did retain significant incremental prediction of global emotion dysregulation even after distress control, demonstrating that it is not entirely reducible to distress. The DERS encompasses a broad range of regulation capacities, including emotional awareness, clarity, impulse control, goal-directed behavior, strategy access, and emotional acceptance; the fact that DIF continues to predict this multifaceted construct even in the presence of depression, anxiety, and stress speaks to its functional significance beyond concurrent mood state.

### 4.3. The Maladaptive Cognitive Regulation Pattern

One finding that warrants specific discussion is the pattern observed for maladaptive cognitive emotion regulation (CERQ maladaptive). At the total-score level, the TAS-20 showed a moderate positive zero-order association with maladaptive cognitive regulation, but in the full regression model it emerged as a small negative predictor after controlling for distress (*β* = −0.08). At the facet level, both DIF and DDF dropped to non-significance, while EOT maintained a significant negative association (*β* = −0.18). Meanwhile, all three DASS-21 subscales were significant positive predictors, with stress showing the largest coefficient (*β* = 0.37).

It should be noted that in the full model, the unique coefficient of each DASS-21 subscale reflects differentiation within the distress space; that is, what is specific to that subscale after removing variance shared with the other two DASS-21 subscales, rather than the total contribution of any single distress dimension. This pattern indicates that the bivariate association between alexithymia and maladaptive cognitive strategies is almost entirely accounted for by shared distress variance. Maladaptive cognitive strategies assessed by the CERQ (i.e., rumination, catastrophizing, self-blame, and blaming others) are theoretically characterized as distress-linked regulatory processes and are largely driven by the motivational pressure of acute negative affect rather than by stable deficits in emotional processing infrastructure. They involve sustained, repetitive engagement with negative emotional content, which requires both the motivation to engage with distressing material (provided by distress itself) and the introspective capacity to engage in such cognitive elaboration. Alexithymia, as a construct thought to reflect deficits in the cognitive processing of emotions, would not be expected to uniquely drive excessive cognitive engagement with emotional content once the distress that motivates such engagement is accounted for.

The results for EOT reveal a notable statistical suppression effect. In the zero-order correlations, EOT showed no significant association with maladaptive cognitive regulation (*r* = −0.04, ns). However, after accounting for the variance shared with distress and other facets of alexithymia in the regression model, EOT was identified as a significant negative predictor (*β* = −0.18, *p* < 0.001). This suggests that the ‘pure’ variance of EOT, representing a pragmatic, non-introspective thinking style, is statistically associated with reduced engagement in the type of sustained, internal cognitive elaboration required for rumination and catastrophizing. Put differently, rumination and catastrophizing are not merely maladaptive; they are cognitively demanding forms of internal elaboration that require the very introspective infrastructure that individuals high in EOT lack. EOT does not so much protect against maladaptive thinking as it may foreclose the capacity for sustained internal cognitive engagement altogether, whether that engagement takes an adaptive or maladaptive form; this is a pattern consistent with a statistical suppression effect and warrants cautious interpretation pending direct replication. This pattern is consistent with the original, affect-deficit model of alexithymia, in that EOT limits cognitive elaboration of emotion rather than amplifying it (Taylor et al., 2023, 2024).

This pattern was not limited to maladaptive cognitive strategies. In the CERQ adaptive model as well, distress variables accounted for the larger share of unique variance, suggesting that the boundary condition applies broadly to cognitive regulation domains, where both adaptive and maladaptive strategies appear more closely tied to current distress levels than to stable alexithymic deficits. This finding does not undermine the predictive discriminant validity of the TAS-20. Rather, it clarifies the boundary conditions: the TAS-20 demonstrates its strongest incremental prediction for outcomes that reflect deficits in regulatory capacity (emotion dysregulation, reduced adaptive strategy use, increased avoidance-oriented strategies) but not for outcomes that are theoretically linked to acute distress states rather than stable regulatory deficits (rumination, catastrophizing). This distinction is theoretically meaningful and maps coherently onto the alexithymia construct as originally conceptualized—a deficit in the cognitive processing and regulation of emotions, not an excess of maladaptive cognitive activity (Sifneos, 1994; Taylor et al., 1997).

More broadly, the results reveal a consistent domain-level dissociation in the relative contributions of alexithymia and distress. Alexithymia emerged as the stronger unique predictor of what individuals do behaviorally—whether engaging approach-oriented or avoidance-oriented strategies to downregulate emotional distress (BERQ), whereas the unique variance of specific distress dimensions—particularly stress—was the stronger predictor of how individuals cognitively elaborate on emotional events (CERQ). This dissociation is theoretically coherent: behavioral regulation strategies such as withdrawal, avoidance, and help-seeking are directly constrained by the stable trait-based deficits that define alexithymia (limited labeling and communication of affects, reduced introspective capacity), whereas cognitive regulation strategies such as rumination and catastrophizing are more state-dependent processes driven by the acute motivational pressure of current distress. The alexithymia construct, as originally formulated, emphasizes deficits in the identification and communication of emotional feelings rather than excesses of maladaptive mental activity (Taylor et al., 1997). The findings of the present study map this distinction.

### 4.4. Integration with Structural Discriminant Validity Evidence

The present findings complement and extend the structural discriminant validity evidence reported in the companion study (Bagby et al., 2026). That study demonstrated that at the subscale level, the TAS-20 DIF subscale loaded 0.863 on the General Emotional Dysfunction factor and only 0.050 on the Psychological Distress factor, a pattern replicated across all TAS-20 and PAQ subscales. The present study demonstrates that this structural distinctiveness has functional consequences: the variance unique to the TAS-20 predicts emotion regulation outcomes that distress alone does not account for.

Together, these two lines of evidence address the discriminant validity question at multiple levels of analysis. Structural discriminant validity establishes that the constructs are psychometrically distinguishable; predictive discriminant validity establishes that the distinction matters in practice. Neither form of evidence alone is sufficient. Demonstrating factor separability without showing that the unique variance predicts meaningful outcomes leaves open the possibility that the statistical distinction is trivial; demonstrating incremental prediction without first establishing structural separability could reflect multicollinearity artifacts or suppressor effects. The convergence of structural and predictive evidence across two studies using the same enriched community sample provides a substantially more compelling case for the discriminant validity of the TAS-20 than either approach in isolation. Indeed, Bagby et al. (2026) explicitly identified the absence of incremental validity analyses as a limitation of the structural approach and called for precisely the kind of predictive tests reported here.

This multi-level approach also reframes the debate initiated by Preece et al. (2020, 2024). Their concern was framed in structural terms: Does the TAS-20 DIF subscale cross-load on distress? Bagby et al.’s (2026) study showed that this cross-loading is not robust across samples. The present study adds a further dimension: even if one were to accept that DIF shares meaningful variance with distress, the TAS-20 as a whole, DDF and EOT in particular, capture small but statistically reliable unique variance that incrementally predicts emotion regulation outcomes. The question is no longer simply whether alexithymia and distress are structurally separable, but whether the unique alexithymic variance does meaningful explanatory work; the present data demonstrate that it does.

### 4.5. Theoretical Implications

The differential facet-level findings illuminate the multidimensional nature of alexithymia and its contribution to emotion regulation in ways that total-score analyses obscure. Three facets of alexithymia relate to emotion regulation through at least partially distinct mechanisms. The association of DIF with broad emotion dysregulation is more substantially shared with concurrent psychological distress than is the case for DDF or EOT, consistent with its role as the facet most proximally linked to undifferentiated emotional arousal. DDF contributes primarily through behavioral regulation pathways; its unique variance, independent of distress, drives individuals away from socially engaged, approach-oriented regulation and toward avoidance and withdrawal. EOT contributes through cognitive regulation pathways; its unique variance constrains the capacity for deliberate, introspective cognitive engagement with emotional material, reducing both adaptive (reappraisal, planning) and maladaptive (rumination, catastrophizing) cognitive strategies.

These patterns are consistent with the distinction between trait-based and state-dependent pathways to emotion regulation difficulty outlined in the Introduction. The stability of DDF and EOT coefficients after distress control supports their characterization as reflecting enduring deficits in emotion processing infrastructure, whereas the attenuation of DIF and the CERQ boundary condition both suggest that distress-driven, state-dependent processes play a larger role in domains involving cognitive engagement with emotional content. More broadly, these differential pathways are consistent with the original theoretical framework (Nemiah et al., 1976; Sifneos, 1973, 1994; Taylor et al., 1997), which emphasized that the components of alexithymia, although intercorrelated, reflect distinct impairments in the cognitive processing and regulation of affect. The present data suggest that these distinct impairments have correspondingly distinct downstream consequences for emotion regulation.

This has implications for intervention: treatments targeting alexithymia may need to address different facets depending on whether the therapeutic goal is improving the identification and differentiation of emotional feelings (DIF), facilitating emotional communication and social engagement (DDF), or expanding the capacity for introspective thinking (EOT).

### 4.6. Strengths, Limitations, and Future Directions

This study has several strengths. It is, to our knowledge, the first to explicitly frame and test the TAS-20 discriminant validity debate in terms of predictive discriminant validity relative to distress, and to examine incremental prediction systematically across multiple emotion regulation domains. The use of a community sample enriched with participants having psychiatric histories provided adequate variance in both alexithymia and distress for detecting meaningful associations. The inclusion of multiple criterion measures spanning global emotion dysregulation (DERS), cognitive regulation strategies (CERQ), and behavioral regulation strategies (BERQ) allowed examination of whether incremental prediction is domain-general or domain-specific. The dual-level analytic approach, examining both total scores and facet scores, revealed patterns that would be invisible to either approach alone. The use of squared semi-partial correlations to compare unique predictor contributions provided a metric that is invariant to entry order, allowing direct comparison of alexithymia and distress contributions within the same model. Finally, the study complements and extends the companion structural analysis paper (Bagby et al., 2026) that used the same participant pool, providing convergent evidence from two distinct methodological approaches.

Several limitations should be acknowledged. First, the cross-sectional design precludes causal inference. Although we interpret the attenuation of DIF after distress control as reflecting shared variance, it is equally possible that distress mediates the DIF–emotion regulation relationship, and cross-sectional regression cannot distinguish these interpretations. Longitudinal designs or experimental paradigms would be needed to establish the causal architecture linking alexithymia facets, distress, and regulation outcomes. It should be noted, however, that the trait stability of the TAS-20 has been extensively documented in the longitudinal literature, with retest reliability coefficients averaging *r* = 0.74 across studies spanning intervals from two weeks to 11 years, and strong evidence of relative stability across medical and psychiatric disorder samples even in the context of changing illness severity (Bagby et al., 2020); the concern that TAS-20 scores primarily reflect transient distress states is, therefore, inconsistent with the accumulated longitudinal evidence.

Second, the exclusive reliance on self-report measures introduces a shared method variance that may inflate associations among predictors and outcomes. Multi-method approaches incorporating performance-based measures of emotion regulation (e.g., behavioral tasks, physiological indices) and alternative alexithymia assessments would strengthen conclusions about incremental prediction. The Toronto Structured Interview for Alexithymia (TSIA; Bagby et al., 2006) would reduce reliance on a single assessment method, though it remains dependent on the individual’s capacity and willingness to report on their own emotional experience. The 20-item Toronto Alexithymia Scale—Informant Form (TAS-20-IF; Bagby et al., 2021) would be particularly informative in this regard, as it bypasses self-report entirely by obtaining ratings from knowledgeable informants. Because the TAS-20-IF assesses the same three facets of alexithymia from an external vantage point, demonstrating that informant-rated alexithymia incrementally predicts emotion regulation outcomes beyond distress would provide evidence for predictive discriminant validity that is free from the shared method variance inherent in self-report designs. That said, given that shared method variance would be expected to inflate correlations among all self-report measures in the model, the fact that the TAS-20 retains substantial unique prediction after controlling for the DASS-21 subscales suggests that the incremental effects reported here are unlikely to be method artifacts. Relatedly, all criterion measures (DERS, CERQ, BERQ) are themselves self-report instruments, meaning that shared method variance operates not only among predictors but also between predictors and outcomes; multi-method criterion assessment (e.g., behavioral tasks, physiological indices of emotion regulation) would provide a more stringent test.

Third, the DASS-21, while widely used, captures only three dimensions of psychological distress. Other operationalizations of distress, including measures with different temporal frameworks, broader symptom coverage, or continuous severity assessments, might yield different patterns of shared variance.

Fourth, while the classification of CERQ and BERQ subscales into adaptive and maladaptive composites follows established conventions (Garnefski et al., 2001; Kraaij & Garnefski, 2019), this binary grouping may obscure strategy-specific associations. Future analyses at the individual subscale level could reveal more granular patterns, for example, whether DDF specifically predicts reduced social support seeking, or whether EOT specifically predicts reduced cognitive reappraisal. Finally, although our sample was enriched with individuals having psychiatric histories, participants were not recruited from clinical treatment settings. It should also be noted that the structural discriminant validity evidence reported in Bagby et al. (2026) and the predictive validity evidence reported here were derived from overlapping participant pools; convergence across the two papers is, therefore, methodologically complementary rather than an independent replication, and replication in independent samples remains an important future priority. Replication in other samples, especially those from actively symptomatic clinical populations, in which both alexithymia and distress are more severe, and their relationship may differ, remains an important priority.

Future research should extend the predictive discriminant validity framework to additional criterion domains. Clinically, alexithymia has been implicated in treatment response, therapeutic alliance, somatization, substance use, and illness behavior. Demonstrating that the TAS-20 retains statistically significant, clinically meaningful predictive capacity of these outcomes after accounting for concurrent distress would further establish the construct validity and clinical utility of alexithymia as a measurement target distinct from psychological distress. The predictive discriminant validity framework employed here should also be applied to other self-report measures of the alexithymia construct. In particular, the Bermond–Vorst Alexithymia Questionnaire (BVAQ; Vorst & Bermond, 2001) assesses alexithymia across five dimensions, including emotionalizing and fantasizing, that extend beyond the three-facet model captured by the TAS-20. Demonstrating that the BVAQ retains incremental prediction of emotion regulation outcomes beyond distress would establish the generalizability of the present findings across different operationalizations of the alexithymia construct while also testing whether the additional dimensions assessed by the BVAQ contribute unique predictive information. Finally, parallel analyses using the Perth Alexithymia Questionnaire (PAQ; Preece et al., 2018) would allow direct comparison of predictive discriminant validity across the most widely discussed alexithymia measures in the current debate.

### 4.7. Clinical and Research Implications

These findings have direct implications for the clinical use and interpretation of the TAS-20. The statistically significant, domain-specific incremental prediction retained after controlling for distress supports the continued use of the TAS-20 in research and clinical assessment contexts, particularly when emotion regulation difficulties are part of the assessment question. The present findings are consistent with the interpretation that elevated TAS-20 scores capture variance in emotion regulation that extends beyond concurrent distress; longitudinal and multi-method validation would be needed before strong clinical inferences about individual patients can be drawn with confidence. The facet-level findings further suggest that clinical interpretation should consider the specific pattern of subscale elevations. Elevated DDF, in particular, may signal impairment in emotion communication that contributes to behavioral withdrawal and avoidance independently of mood state, with implications for treatment planning that target emotional labeling and interpersonal expression. Elevated EOT may signal a concrete thinking style that limits the capacity for cognitive emotion regulation strategies, with implications for the selection and adaptation of cognitive–behavioral interventions.

For researchers, the present findings underscore that the discriminant validity question cannot be resolved by structural analyses alone. The field’s focus on factor analytic separability, while necessary, is not sufficient to establish that a measurement distinction has practical significance. We recommend that future evaluations of the validity of alexithymia measures routinely include tests of incremental predictive validity alongside structural analyses, providing a more complete evidentiary basis for evaluating construct and measure utility.

## 5. Conclusions

This study supports the predictive discriminant validity of the TAS-20 in the context of the ongoing debate about its relationship with psychological distress. Using hierarchical multiple regression in a community sample enriched with psychiatric histories, we demonstrated that the TAS-20 retains statistically significant, domain-specific incremental prediction of emotion regulation outcomes after controlling for depression, anxiety, and stress, although the magnitude of these increments was modest and did not reach the 0.15 heuristic proposed by Hunsley and Meyer (2003) for clinically meaningful incremental validity. At the total-score level, the TAS-20 accounted for a larger share of unique variance than any individual distress subscale in global emotion dysregulation, adaptive behavioral regulation, and maladaptive behavioral regulation, while retaining smaller unique contributions to cognitive regulation outcomes. At the facet level, DDF and EOT emerged as the most consistent unique predictors across outcomes after distress control, whereas DIF showed greater attenuation—a pattern consistent with the theoretically expected proximity of emotion identification deficits to affective disturbance.

These predictive findings complement the structural discriminant validity evidence from our companion study (Bagby et al., 2026), jointly demonstrating that alexithymia as measured by the TAS-20 is not only statistically separable from psychological distress but also functionally separable in its associations with emotion regulation. The variance captured by the TAS-20 does explanatory work that distress measures do not—most clearly for global emotion dysregulation and behavioral regulation outcomes, and to a smaller but statistically reliable degree for cognitive regulation strategies—supporting the continued utility of this instrument in clinical and research settings. The present findings are inconsistent with the claim that the TAS-20 primarily measures psychological distress; however, questions about the nature and extent of construct overlap, particularly with respect to the DIF subscale, remain theoretically open and warrant continued investigation. The results from this study indicate that the TAS-20 measures a multifaceted personality trait whose components relate to emotion regulation through partially distinct pathways, which remain operative and measurable even in the context of concurrent psychological distress.

## Figures and Tables

**Table 1:** Descriptive statistics and internal consistency estimates for all study variables.

Measures	*M*	*SD*	Range	Skewness	Kurtosis	α	ω^t^
**DERS**							
Total	81.00	26.07	36–157	0.57	−0.27	0.96	0.95
Non-Acceptance	12.67	6.08	6–30	0.82	−0.19	0.93	0.93
Goals	13.57	5.39	5–25	0.32	−0.84	0.91	0.92
Impulse	10.59	4.89	6–29	1.27	0.94	0.88	0.88
Awareness	15.49	5.53	6–30	0.45	−0.45	0.87	0.87
Strategies	17.90	7.81	8–40	0.73	−0.36	0.92	0.93
Clarity	13.21	5.22	6–30	0.91	0.33	0.87	0.87
**TAS-20**							
Total	45.58	13.56	20–83	0.50	−0.35	0.90	0.90
DIF	14.47	6.72	7–34	0.80	−0.30	0.91	0.91
DDF	12.44	5.02	5–25	0.43	−0.64	0.84	0.84
EOT	18.68	4.65	8–31	0.14	−0.43	0.64	0.63
**DASS-21**							
Total	14.32	12.96	0–59	0.99	0.33	0.95	0.95
Stress	5.40	4.83	0–21	0.84	0.07	0.89	0.89
Anxiety	3.53	4.06	0–20	1.46	1.73	0.84	0.85
Depression	5.40	5.65	0–21	0.97	−0.03	0.93	0.93
**BERQ**							
*Adaptive*	33.38	8.55	12–60	0.16	−0.46	0.86	0.84
Distraction	11.54	3.46	4–20	0.22	−0.55	0.81	0.81
Approaching	12.11	3.99	4–20	0.26	−0.74	0.90	0.90
Social Support	9.74	4.16	4–20	0.53	−0.53	0.90	0.90
*Maladaptive*	19.59	7.60	8–40	0.57	−0.19	0.90	0.90
Withdrawal	10.65	4.71	4–20	0.43	−0.96	0.93	0.93
Ignoring	8.95	3.95	4–20	0.75	−0.16	0.87	0.87
**CERQ**							
*Adaptive*	61.56	14.32	21–100	0.00	−0.51	0.91	0.92
Acceptance	12.57	3.33	4–20	0.01	−0.57	0.74	0.74
Planning	13.34	3.74	4–20	−0.24	−0.58	0.82	0.83
Perspective	12.71	3.84	4–20	−0.09	−0.71	0.82	0.82
Refocusing	10.49	3.86	4–20	0.25	−0.63	0.85	0.85
Reappraisal	12.38	4.14	4–20	−0.07	−0.89	0.87	0.87
*Maladaptive*	38.69	10.15	16–73	0.48	0.21	0.87	0.86
Self-Blame	10.78	3.68	4–20	0.51	−0.38	0.82	0.83
Rumination	11.57	3.62	4–20	0.10	−0.64	0.74	0.73
Catastrophizing	8.47	3.49	4–20	0.89	0.33	0.79	0.79
Blaming	7.92	2.89	3–19	0.98	1.20	0.81	0.81

Note. N = 661. *M* = mean; *SD* = standard deviation; α = coefficient alpha; ω^t^ = coefficient omega; DERS = Difficulties in Emotion Regulation Scale; TAS-20 = 20-item Toronto Alexithymia Scale; DASS-21 = Depression Anxiety Stress Scale 21-item; DIF = difficulty identifying feelings; DDF = difficulty describing feelings; EOT = externally oriented thinking; BERQ = Behavioral Emotion Regulation Questionnaire; CERQ = Cognitive Emotion Regulation Questionnaire.

**Table 2:** Hierarchical regression: TAS-20 total score predicting DERS total after controlling for psychological distress.

Predictor	B	*SE*	*β*	*sr* ^2^	95% CI		*p*
					*LL*	*UL*	
**Step 1**							
DASS-21 Stress	2.12	0.22	0.39	0.06	1.69	2.54	<0.001
DASS-21 Anxiety	0.09	0.24	0.01	0.00	−0.38	0.56	0.71
DASS-21 Depression	2.05	0.17	0.45	0.10	1.72	2.38	<0.001
**Step 2**							
DASS-21 Stress	1.69	0.19	0.31	0.04	1.32	2.06	<0.001
DASS-21 Anxiety	−0.15	0.21	−0.02	0.00	−0.56	0.25	0.46
DASS-21 Depression	1.45	0.15	0.32	0.04	1.16	1.74	<0.001
TAS-20 Total Score	0.75	0.05	0.39	0.10	0.65	0.85	<0.001

Note. N = 602. TAS-20 = Toronto Alexithymia Scale 20-item; DERS = Difficulties in Emotion Regulation Scale; DASS-21 = Depression Anxiety Stress Scale 21-item; Total Score = total raw score. Model results in Step 1: *F*(3, 598) = 329.388, *p* < 0.001, *R*^2^ = 0.62. Model results in Step 2: *F*(4, 597) = 390.26, *p* < 0.001, *R*^2^ = 0.72; Δ*R*^2^ = 0.10.

**Table 3:** Hierarchical regression: TAS-20 total score predicting BERQ adaptive and maladaptive composite after controlling for psychological distress.

Predictor	B	*SE*	*β*	*sr* ^2^	95% CI		*p*
					*LL*	*UL*	
**BERQ Adaptive**							
**Step 1**							
DASS-21 Stress	0.09	0.11	0.05	0.00	−0.12	0.30	0.41
DASS-21 Anxiety	0.41	0.12	0.19	0.02	0.17	0.65	<0.001
DASS-21 Depression	−0.66	0.09	−0.43	0.08	−0.82	−0.49	<0.001
**Step 2**							
DASS-21 Stress	0.24	0.11	0.13	0.01	0.03	0.45	0.03
DASS-21 Anxiety	0.46	0.12	0.22	0.02	0.23	0.69	<0.001
DASS-21 Depression	−0.49	0.08	−0.32	0.04	−0.65	−0.32	<0.001
TAS-20 Total Score	−0.22	0.03	−0.35	0.08	−0.28	−0.17	<0.001
**BERQ Maladaptive**							
**Step 1**							
DASS-21 Stress	0.35	0.08	0.22	0.02	0.19	0.50	<0.001
DASS-21 Anxiety	−0.04	0.09	−0.02	0.00	−0.20	0.13	0.67
DASS-21 Depression	0.64	0.06	0.48	0.10	0.52	0.76	<0.001
**Step 2**							
DASS-21 Stress	0.23	0.07	0.15	0.01	0.09	0.38	0.002
DASS-21 Anxiety	−0.09	0.08	−0.05	0.00	−0.25	0.06	0.25
DASS-21 Depression	0.49	0.06	0.37	0.06	0.37	0.60	<0.001
TAS-20 Total Score	0.19	0.02	0.33	0.07	0.15	0.22	<0.001

Note. BERQ adaptive N = 624. BERQ maladaptive N = 630. BERQ = Behavioral Emotion Regulation Questionnaire; TAS-20 = 20-item Toronto Alexithymia Scale; DASS-21 = Depression Anxiety Stress Scale 21-item; Total Score = total raw score. BERQ adaptive model results in Step 1: *F*(3, 620) = 23.15, *p* < 0.001, *R*^2^ = 0.10. BERQ adaptive model results in Step 2: *F*(4, 619) = 34.17, *p* < 0.001, *R*^2^ = 0.18; Δ*R*^2^ = 0.08. BERQ Maladaptive model results in Step 1: *F*(3, 626) = 144.03, *p* < 0.001, *R*^2^ = 0.41. Maladaptive model results in Step 2: *F*(4, 625) = 144.52, *p* < 0.001, *R*^2^ = 0.48; Δ*R*^2^ = 0.07.

**Table 4:** Hierarchical regression: TAS-20 total score predicting CERQ adaptive and maladaptive composites after controlling for psychological distress.

Predictor	B	*SE*	*β*	*sr* ^2^	95% CI		*p*
					*LL*	*UL*	
**CERQ Adaptive**							
**Step 1**							
DASS-21 Stress	−0.10	0.19	−0.03	0.00	−0.47	0.27	0.61
DASS-21 Anxiety	0.79	0.21	0.23	0.02	0.39	1.20	<0.001
DASS-21 Depression	−0.87	0.15	−0.34	0.05	−01.16	−0.58	<0.001
**Step 2**							
DASS-21 Stress	0.00	0.19	0.00	0.00	−0.37	0.38	0.98
DASS-21 Anxiety	0.84	0.21	0.24	0.03	0.44	1.24	<0.001
DASS-21 Depression	−0.74	0.15	−0.29	0.04	−1.04	−0.45	<0.001
TAS-20 Total Score	−0.16	0.05	−0.15	0.02	−0.26	−0.06	0.002
**CERQ Maladaptive**							
**Step 1**							
DASS-21 Stress	0.81	0.11	0.38	0.06	0.59	1.02	<0.001
DASS-21 Anxiety	0.36	0.12	0.14	0.01	0.12	0.59	0.003
DASS-21 Depression	0.26	0.08	0.14	0.01	0.09	0.42	0.002
**Step 2**							
DASS-21 Stress	0.84	0.11	0.4	0.06	0.63	1.06	<0.001
DASS-21 Anxiety	0.38	0.12	0.15	0.01	0.14	0.61	0.002
DASS-21 Depression	0.31	0.09	0.17	0.01	0.14	0.48	<0.001
TAS-20 Total Score	−0.06	0.03	−0.08	0.01	−0.12	−0.01	0.03

Note. CERQ adaptive N = 614; CERQ maladaptive N = 624. CERQ = Cognitive Emotion Regulation Questionnaire; TAS-20 = 20-item Toronto Alexithymia Scale; Total Score = total raw score. CERQ adaptive model results in Step 1: *F*(3, 610) = 15.80, *p* < 0.001, *R*^2^ = 0.07. CERQ adaptive model results in Step 2: *F*(4, 609) = 14.54, *p* < 0.001, *R*^2^ = 0.09; Δ*R*^2^ = 0.02. CERQ Maladaptive model results in Step 1: *F*(3, 620) = 121.53, *p* < 0.001, *R*^2^ = 0.37. Maladaptive model results in Step 2: *F*(4, 619) = 92.88, *p* < 0.001, *R*^2^ = 0.38; Δ*R*^2^ = 0.01.

**Table 5:** Hierarchical regression: TAS-20 subscales predicting DERS total after controlling for psychological distress.

Predictor	B	*SE*	*β*	*sr* ^2^	95% CI		*p*
					*LL*	*UL*	
**Step 1**							
DASS-21 Stress	2.12	0.22	0.39	0.06	1.69	2.54	<0.001
DASS-21 Anxiety	0.09	0.24	0.01	0.00	−0.38	0.56	0.71
DASS-21 Depression	2.05	0.17	0.45	0.10	1.72	2.38	<0.001
**Step 2**							
DASS-21 Stress	1.61	0.19	0.3	0.03	1.23	1.98	<0.001
DASS-21 Anxiety	−0.25	0.21	−0.04	0.00	−0.66	0.15	0.22
DASS-21 Depression	1.43	0.15	0.31	0.04	1.14	1.72	<0.001
TAS-20 DIF	0.89	0.14	0.23	0.02	0.61	1.16	<0.001
TAS-20 DDF	0.95	0.19	0.18	0.01	0.58	1.32	<0.001
TAS-20 EOT	0.33	0.14	0.06	0.00	0.06	0.59	0.02

Note. N = 602. TAS-20 = 20-item Toronto Alexithymia Scale; DERS = Difficulties in Emotion Regulation Scale; DASS-21 = Depression Anxiety Stress Scale 21-item; DIF = difficulty identifying feelings; DDF = difficulty describing feelings; EOT = externally oriented thinking. Model results in Step 1: *F*(3, 598) = 329.39, *p* < 0.001, *R*^2^ = 0.62. Model results in Step 2: *F*(6, 595) = 266.14, *p* < 0.001, *R*^2^ = 0.73; Δ*R*^2^ = 0.11.

**Table 6:** Hierarchical regression: TAS-20 subscales predicting BERQ adaptive and maladaptive composites after controlling for psychological distress.

Predictor	B	*SE*	*β*	*sr* ^2^	95% CI		*p*
					*LL*	*UL*	
**BERQ Adaptive**							
**Step 1**							
DASS-21 Stress	0.09	0.11	0.05	0.00	−0.12	0.30	0.41
DASS-21 Anxiety	0.41	0.12	0.19	0.02	0.17	0.65	<0.001
DASS-21 Depression	−0.66	0.09	−0.43	0.08	−0.82	−0.49	<0.001
**Step 2**							
DASS-21 Stress	0.20	0.11	0.11	0.00	−0.01	0.40	0.06
DASS-21 Anxiety	0.40	0.12	0.19	0.02	0.17	0.63	<0.001
DASS-21 Depression	−0.48	0.08	−0.31	0.04	−0.65	−0.32	<0.001
TAS-20 DIF	0.02	0.08	0.02	0.00	−0.14	0.18	0.80
TAS-20 DDF	−0.43	0.10	−0.25	0.02	−0.64	−0.23	<0.001
TAS-20 EOT	−0.31	0.08	−0.17	0.02	−0.46	−0.17	<0.001
**BERQ Maladaptive**							
**Step 1**							
DASS-21 Stress	0.35	0.08	0.22	0.02	0.19	0.50	<0.001
DASS-21 Anxiety	−0.04	0.09	−0.02	0.00	−0.20	0.13	0.67
DASS-21 Depression	0.64	0.06	0.48	0.10	0.52	0.76	<0.001
**Step 2**							
DASS-21 Stress	0.24	0.07	0.15	0.01	0.09	0.38	0.001
DASS-21 Anxiety	−0.07	0.08	−0.04	0.00	−0.23	0.09	0.37
DASS-21 Depression	0.48	0.06	0.36	0.05	0.36	0.59	<0.001
TAS-20 DIF	−0.01	0.05	−0.01	0.00	−0.12	0.10	0.88
TAS-20 DDF	0.51	0.07	0.33	0.04	0.36	0.65	<0.001
TAS-20 EOT	0.09	0.05	0.05	0.00	−0.02	0.19	0.10

Note. BERQ adaptive N = 624; BERQ maladaptive N = 630. BERQ = Behavioral Emotion Regulation Questionnaire*;* TAS-20 = 20-item Toronto Alexithymia Scale; DASS-21 = Depression Anxiety Stress Scale 21-item; DIF = difficulty identifying feelings; DDF = difficulty describing feelings; EOT = externally oriented thinking. BERQ adaptive model results in Step 1: *F*(3, 620) = 23.15, *p* < 0.001, *R*^2^ = 0.10. BERQ adaptive model results in Step 2: *F*(6, 617) = 25.04, *p* < 0.001, *R*^2^ = 0.20; Δ*R*^2^ = 0.10. BERQ Maladaptive model results in Step 1: *F*(3, 626) = 144.03*, p* < 0.001, *R*^2^ = 0.41. Maladaptive model results in Step 2: *F*(6, 623) = 102.84, *p* < 0.001, *R*^2^ = 0.50; Δ*R*^2^ = 0.09.

**Table 7:** Hierarchical regression: TAS-20 subscales predicting CERQ adaptive and maladaptive composites after controlling for psychological distress.

Predictor	B	*SE*	*β*	*sr* ^2^	95% CI		*p*
					*LL*	*UL*	
**CERQ Adaptive**							
**Step 1**							
DASS-21 Stress	−0.10	0.19	−0.03	0.00	−0.47	0.27	0.61
DASS-21 Anxiety	0.79	0.21	0.23	0.02	0.39	1.20	<0.001
DASS-21 Depression	−0.87	0.15	−0.34	0.05	−1.16	−0.58	<0.001
**Step 2**							
DASS-21 Stress	−0.03	0.19	−0.01	0.00	−0.40	0.34	0.87
DASS-21 Anxiety	0.80	0.21	0.23	0.02	0.39	1.21	<0.001
DASS-21 Depression	−0.75	0.15	−0.29	0.04	−1.05	−0.45	<0.001
TAS-20 DIF	−0.13	0.14	−0.06	0.00	−0.41	0.15	0.36
TAS-20 DDF	−0.03	0.19	−0.01	0.00	−0.39	0.34	0.88
TAS-20 EOT	−0.36	0.14	−0.11	0.01	−0.63	−0.09	0.01
**CERQ Maladaptive**							
**Step 1**							
DASS-21 Stress	0.81	0.11	0.38	0.06	0.59	1.02	<0.001
DASS-21 Anxiety	0.36	0.12	0.14	0.01	0.12	0.59	0.003
DASS-21 Depression	0.26	0.08	0.14	0.01	0.09	0.42	0.002
**Step 2**							
DASS-21 Stress	0.78	0.11	0.37	0.05	0.56	0.99	<0.001
DASS-21 Anxiety	0.29	0.12	0.12	0.01	0.06	0.53	0.01
DASS-21 Depression	0.30	0.09	0.17	0.01	0.13	0.47	<0.001
TAS-20 DIF	0.06	0.08	0.04	0.00	−0.10	0.22	0.46
TAS-20 DDF	0.08	0.11	0.04	0.00	−0.13	0.29	0.48
TAS-20 EOT	−0.39	0.08	−0.18	0.03	−0.54	−0.24	<0.001

Note. CERQ adaptive N = 614; CERQ maladaptive N = 624. CERQ = Cognitive Emotion Regulation Questionnaire; TAS-20 = 20-item Toronto Alexithymia Scale; DASS-21 = Depression Anxiety Stress Scale 21-item; DIF = difficulty identifying feelings; DDF = difficulty describing feelings; EOT = externally oriented thinking. CERQ adaptive model results in Step 1: *F*(3, 610) = 15.80, *p* < 0.001, *R*^2^ = 0.07. CERQ adaptive model results in Step 2: *F*(6, 607) = 10.11, *p* < 0.001, *R*^2^ = 0.09; Δ*R*^2^ = 0.02. CERQ Maladaptive model results in Step 1: *F*(3, 620) = 121.53, *p* < 0.001, *R*^2^ = 0.37. Maladaptive model results in Step 2: *F*(6, 617) = 67.65, *p* < 0.001, *R*^2^ = 0.40; Δ*R*^2^ = 0.03.

## Data Availability

The data presented in this study are available on request from the corresponding author.

## Supplementary Materials

The following supporting information can be downloaded at: https://www.mdpi.com/article/10.3390/bs16060854/s1, Table S1. Zero-Order Correlations Among Alexithymia, Psychological Distress, and Emotion Regulation Variables.
