# Supplementary material for: Predictive Discriminant Validity of the 20-Item Toronto Alexithymia Scale: Incremental Prediction of Emotion Regulation Beyond Psychological Distress
Source: Behav Sci (Basel). 2026 May 27;16(6):854. doi: 10.3390/bs16060854 (PMC13296069; doi:10.3390/bs16060854)
Supplement: Supplementary file 1 [file behavsci-16-00854-s001.zip › behavsci-4218153-supplementary.pdf]

Table S1. Zero-Order Correlations Among Alexithymia, Psychological Distress, and Emotion Regulation Variables.

	1	2	3	4	5	6	7	8	9	10	11	12	13	14	15	16	17	18	19	20	21	22	23	24	25	26	27	28	29	30	31	32
**DERS**																																
1. Total	--																															
2. Non-Acceptance	.79	--																														
3. Goals	.77	.52	--																													
4. Impulsive	.76	.56	.57	--																												
5. Awareness	.53	.25	.18	.20	--																											
6. Strategies	.88	.65	.75	.66	.26	--																										
7. Clarity	.83	.58	.58	.51	.58	.62	--																									
**TAS-20**																																
8. Total	.72	.52	.42	.46	.62	.51	.80	--																								
9. DIF	.72	.55	.47	.51	.45	.56	.80	.89	--																							
10. DDF	.68	.51	.41	.41	.56	.50	.77	.90	.77	--																						
11. EOT	.32	.17	.10	.17	.56	.14	.34	.66	.31	.44	--																					
**DASS-21**																																
12. Total	.77	.60	.64	.62	.26	.77	.62	.58	.63	.55	.17	--																				
13. Stress	.72	.59	.61	.63	.20	.70	.56	.53	.59	.51	.14	.90	--																			
14. Anxiety	.59	.49	.46	.55	.14	.57	.46	.47	.55	.45	.10	.87	.72	--																		
15. Depression	.74	.52	.61	.49	.33	.76	.62	.55	.57	.52	.21	.90	.71	.65	--																	
**BERQ**																																
16. Adaptive Composite	-.29	-.04	-.12	-.08	-.50	-.23	-.33	-.35	-.24	-.34	-.31	-.18	-.11	-.05	-.27	--																
17. Distraction	.02	.18	.05	.03	-.12	-.05	.03	.04	.05	.05	-.02	.05	.07	.08	.00	.66	--															
18. Approaching	-.46	-.20	-.29	-.26	-.48	-.38	-.45	-.43	-.36	-.39	-.29	-.34	-.25	-.21	-.40	.79	.34	--														
19. Social Support	-.16	-.03	.00	.06	-.47	-.06	-.26	-.34	-.18	-.34	-.34	-.08	-.04	.03	-.17	.75	.21	.39	--													
20. Maladaptive Composite	.71	.59	.50	.45	.41	.59	.63	.60	.55	.61	.28	.61	.55	.45	.63	-.18	.27	-.29	-.30	--												
21. Withdrawal	.70	.54	.59	.43	.34	.66	.60	.52	.52	.54	.17	.64	.57	.46	.67	-.25	.11	-.32	-.28	.90	--											
22. Ignoring	.52	.48	.26	.34	.39	.35	.49	.52	.43	.52	.34	.41	.39	.31	.40	-.06	.39	-.18	-.24	.85	.54	--										
**CERQ**																																
23. Adaptive Composite	-.24	.00	-.13	-.10	-.36	-.25	-.19	-.19	-.14	-.15	-.18	-.14	-.10	-.02	-.21	.68	.53	.63	.35	-.03	-.15	.12	--									
24. Acceptance	.30	.33	.27	.15	.07	.32	.28	.22	.24	.24	.05	.30	.26	.21	.32	.09	.19	.06	-.03	.38	.35	.31	.43	--								
25. Planning	-.36	-.11	-.18	-.17	-.46	-.34	-.32	-.31	-.23	-.28	-.26	-.23	-.17	-.08	-.32	.72	.45	.73	.40	-.17	-.26	-.03	.84	.17	--							
26. Perspective	-.21	-.01	-.11	-.14	-.24	-.27	-.14	-.16	-.14	-.12	-.14	-.16	-.12	-.08	-.19	.49	.39	.48	.23	-.04	-.12	.06	.83	.26	.60	--						
27. Refocusing	-.20	-.01	-.13	-.04	-.29	-.21	-.19	-.13	-.11	-.13	-.08	-.12	-.09	.01	-.21	.58	.57	.42	.33	-.03	-.18	.15	.76	.10	.62	.50	--					
28. Reappraisal	-.40	-.16	-.29	-.18	-.40	-.41	-.33	-.31	-.26	-.27	-.22	-.29	-.23	-.11	-.38	.62	.37	.65	.35	-.22	-.33	-.03	.87	.15	.74	.72	.60	--				
29. Maladaptive Composite	.58	.56	.52	.56	-.03	.66	.37	.30	.40	.33	-.04	.60	.59	.51	.51	.15	.18	-.01	.18	.49	.50	.34	.18	.45	.09	.03	.13	-.02	--			
30. Self-Blame	.50	.53	.35	.33	.17	.49	.40	.34	.39	.38	.01	.47	.42	.38	.46	-.03	.10	-.06	-.07	.48	.48	.34	.11	.47	-.02	.06	-.03	-.06	.69	--		
31. Rumination	.31	.38	.39	.37	-.32	.43	.15	.04	.20	.10	-.28	.38	.42	.34	.27	.32	.20	.16	.33	.23	.28	.11	.29	.38	.25	.12	.21	.15	.80	.45	--	
32. Catastrophizing	.59	.47	.49	.58	.10	.65	.36	.36	.39	.34	.12	.59	.56	.49	.51	.00	.08	-.16	.11	.47	.45	.36	.01	.30	-.05	-.09	.07	-.13	.83	.41	.52	--
33. Blaming Others	.30	.24	.29	.40	-.05	.35	.16	.16	.20	.15	.02	.32	.34	.30	.23	.14	.13	.02	.16	.26	.25	.20	.09	.17	.08	-.02	.14	-.03	.63	.09	.35	.54

Note. $|r| \geq .08$, $p < .05$; $|r| \geq .11$, $p < .01$; $|r| \geq .14$, $p < .05$. Two-tailed correlations. N = 602-660.
